# Protocol for the induction of innate immune memory in human smooth muscle cells and endothelial cells *in vitro*

**DOI:** 10.1016/j.xpro.2023.102776

**Published:** 2023-12-19

**Authors:** Jannik Sonnenberg, Dennis Schwarz, Sina MM. Lagache, Lucia Schnack, Helena Körner, Merle Leffers, Hannah Hardege, Yuanyuan Liu, Holger Reinecke, Hannes M. Findeisen, Yahya Sohrabi

**Affiliations:** 1 Department of Cardiology I - Coronary and Peripheral Vascular Disease, Heart Failure, University Hospital Münster, Münster, Germany; 2 Department of Medical Genetics, Third Faculty of Medicine, Charles University, Prague, Czechia; 3 Institutes of Molecular Genetics of the Czech Academy of Sciences, Prague, Czechia

**Keywords:** Cell Biology, Immunology, Molecular Biology

## Abstract

Non-immune cells, like innate immune cells, can develop a memory-like phenotype in response to priming with microbial compounds or certain metabolites, which enables an enhanced response to a secondary unspecific stimulus. This paper describes a step-by-step protocol for the induction and analysis of trained immunity in human endothelial and smooth muscle cells. We then describe steps for cell culture with cryopreserved vascular cells, subcultivation, and induction of trained immunity. We then provide detailed procedures for downstream analysis using ELISA and qPCR.

For complete details on the use and execution of this protocol, please refer to Sohrabi et al. (2020)^1^ and Shcnack et al.^2^

## Highlights

• Instructions on culturing of cryopreserved vascular cells
• Detailed steps for endothelial cells culture and subculture
• Detailed steps for smooth muscle cells culture and subculture
• Analysis of *in vitro* innate immune memory induced in vascular cells

## Abstract (editor highlights)

Publisher’s note: Undertaking any experimental protocol requires adherence to local institutional guidelines for laboratory safety and ethics.

## Graphical abstract

**Figure:**
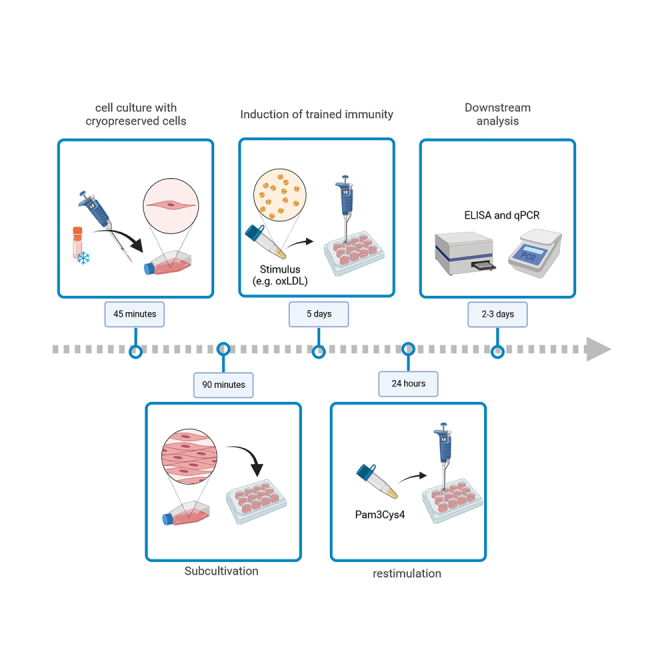

## Before you begin

Innate immune memory/trained immunity is a newly described phenomenon that has evolved very fast during the last decade.^3^ Growing evidence shows that innate immune cells as well as vascular cells can develop a memory-like phenotype in response to different inflammatory stimuli.^1^^,^^2^ The memory is induced through epigenetic modifications in the histones and cellular metabolic changes, which enables the trained cells to exhibit enhanced inflammatory reactions in response to a secondary stimulus.^4^^,^^5^ Although development of innate immune memory is beneficial in improving host responses to an infection, it can contribute in inflammatory diseases onset and development. Therefore, current knowledge suggests trained immunity as an approach to prevent and treat the diseases. The protocol below states the step-by-step instructions for inducing trained immunity in vascular cells. All procedures are performed in a Class I biological safety hood with standard aseptic technique and cells are cultured in a humidified 37°C incubator with 5% CO_2_.

### General laboratory preparations prior to starting the work

**Timing: 1 h**

1. Make sure that all reagents are available.
2. Start the cell culture laminar flow bench and carefully disinfect the surface and the pipettes. Make sure that all required consumables are sterile.
3. Prepare media and solutions.
4. Set the water bath at 37°C and prewarm medium.

## Key resources table

**Table:** 

REAGENT or RESOURCE	SOURCE	IDENTIFIER
**Chemicals, peptides, and recombinant proteins**		

Human vascular smooth muscle cell basal medium	Thermo Fisher Scientific	Cat.no.: M231500
Smooth muscle growth supplement	Thermo Fisher Scientific	Cat.no.: S00725
EGM-2 Endothelial SingleQuots Kit	Lonza	Cat.no.: CC-4176
EBM-2 endothelial cell growth basal medium-2	Lonza	Cat.no.: CC-3156
RPMI-1640 medium	Thermo Fisher Scientific	Cat.no.: 21875034
Penicillin-Streptomycin	Sigma-Aldrich	Cat.no.: P4333
Accutase	PAN-Biotech	Cat.no.: P10-2110M
Fetal bovine serum	Sigma-Aldrich	Cat.no.: F7524-500ML
Bovine serum albumin	AppliChem	CAS 9048-46-8
Hydrochloric acid 37%	Roth	CAS 7647-01-0
oxLDL	House made	https://www.frontiersin.org/articles/10.3389/fimmu.2018.03155/full
Pam3Cys-SKKKK (Pam3Cys4)	EMC	Cat.no.: L2000
Heparin-Natrium	Ratiopharm	Cat.no.: 5394.02.00
Tween 20	Sigma-Aldrich	CAS 9005-64-5
Dulbecco’s phosphate-buffered saline (PBS)	Serva	Cat.no.: 47302

**Critical commercial assays**		

Human IL-6 DuoSet ELISA	R&D	Cat.no.: DY206
Human IL-8 DuoSet ELISA	R&D	Cat.no.: DY208

**Deposited data**		

Raw and analyzed data	This paper, Schnack et al.^2^	https://www.frontiersin.org/articles/10.3389/fimmu.2019.00013/full

**Experimental models: Cell lines**		

Human coronary artery smooth muscle cells	Lonza	Cat.no.: CC-2583
Human aortic endothelial cells	PromoCell	Cat.no.: C-12271
Human umbilical vein endothelial cells	PromoCell	Cat.no.: C-12200

**Software and algorithms**		

WorkOut 2.5	Dazdaq Solutions	https://www.myassays.com/download-and-install-workout-25.html

**Other**		

VICTOR X3 multimode plate reader	PerkinElmer	Part number 2030-0050
Neubauer hemocytometer	Marienfeld	Cat.no.: 0640110
96-well plate	Greiner Bio-One	Cat.no.: #655061

## Materials and equipment

**Table:** Smooth muscle cell growth medium (SMGM)

Reagent	Catalog #	Final concentration in the medium	Amount
Human Vascular Smooth Muscle Cell Basal Medium	Gibco: #M231500	N/A	500 mL
Smooth Muscle Growth Supplement (SMGS)	Gibco: #S00725	5%	25 mL
Penicillin-Streptomycin	Gibco: # 15140122	1%	5 mL
**Total**		**N/A**	**530 mL**

Store at 4°C; protect from light; do not freeze; stable until expiration date.

***Alternatives:*** Replace the Human Vascular Smooth Muscle Cell Basal Medium and Smooth Muscle Growth Supplement with Smooth Muscle Cell Growth Medium 2 (# C-22062) from PromoCell GmbH.

**Table:** Endothelial cell growth medium (ECGM)

Reagent	Catalog #	Final concentration in the medium	Amount
EBM-2 Endothelial Cell Growth Basal Medium-2	Lonza: # 00190860	N/A	500 mL
EGM-2 Endothelial SingleQuots Kit	Lonza: # CC-4176	N/A	5,2 mL
Fetal Bovine Serum	Sigma: #F7524	5%	25 mL
Penicillin-Streptomycin	Gibco: # 15140122	1%	5 mL
**Total**		**N/A**	**535,2 mL**

Store at 4°C; protect from light; do not freeze; stable until expiration date.

***Alternatives:*** Alternatively, the EBM-2 Endothelial Cell Growth Medium-2 (Lonza) can be replaced with EGM-2 Endothelial cells medium (PromoCell #22111).

***Note:*** The amount of serum may vary depending on the cell type, for example, if using human umbilical vein endothelial cells (HUVECs), the amount of serum can be reduced to 2%, whereas human aortic endothelial cells (HAECs) require 5% serum.

***Alternatives:*** M199 medium (Gibco: # 21180–021) supplemented with Bovine Pituitary Extract (Gibco: # 13028–014), 30 μg/mL Heparin, 20% FBS, and antibiotics can also be used for culturing endothelial cells.

**Table:** Smooth muscle cell resting medium (SMRM)

Reagent	Catalog #	Final concentration	Amount
Human Vascular Smooth Muscle Cell Basal Medium	Gibco: #M231500	N/A	500 mL
Fetal Bovine Serum	Sigma: #F7524	0.4%	2 mL
Penicillin-Streptomycin	Gibco: # 15140122	1%	5 mL
Heparin	Ratiopharm	30 μg/mL	1.5 mL
**Total**		**N/A**	**508.5 mL**

Store at 4°C; protect from light; do not freeze; stable until expiration date.

***Alternatives:*** Replace the Human Vascular Smooth Muscle Cell Basal Medium with Smooth Muscle Cell Basal Medium 2 from PromoCell.

**Table:** Endothelial cell resting medium (ECRM)

Reagent	Catalog #	Final concentration	Amount
EBM-2 Endothelial Cell Growth Basal Medium-2	Lonza: # 00190860	N/A	500 mL
EGM-2 Endothelial SingleQuots Kit excluding VEGF and EGF	Lonza: # CC-4176	N/A	0,52 mL
Fetal Bovine Serum	Sigma: #F7524	5%	25 mL
Penicillin-Streptomycin	Gibco: # 15140122	1%	5 mL
**Total**		**N/A**	**530,52 mL**

Store at 4°C; protect from light; do not freeze; stable until expiration date.

***Alternatives:*** Replace the EBM-2 Endothelial Cell Growth Basal Medium-2 and EGM-2 Endothelial Basal Medium (PromoCell #22211).

***Note:*** If using HUVECs, the amount of serum can be decreased to 2%.

***Alternatives:*** M199 medium (Gibco: # 21180–021) supplemented with Bovine Pituitary Extract (Gibco: # 13028–014), 30 μg/mL Heparin, 5% FBS, and antibiotics can also be used as resting medium.

**Table:** Detachment solution

Reagent	Final concentration	Amount
Accutase	50%	2.5 mL
Phosphate-buffered saline	50%	2.5 mL
**Total**	**N/A**	**5 mL**

Store at -20°C; protect from light; Accutase is stable for 1 year from date of production.

***Alternatives:*** Trypsin-EDTA 0.25% (Gibco: # 25200056) diluted 1:4 with PBS can also be used to detach cells.

**Table:** Detachment stop medium

Reagent	Final concentration	Amount
RPMI-1640 Medium	N/A	9 mL
FBS	10%	1 mL
**Total**	**N/A**	**10 mL**

Store at 4°C; protect from light; do not freeze; stable until expiration date.

***Alternatives:*** Replace the RPMI-1640 Medium with any other basal buffered medium such as DMEM.

**Table:** ELISA washing buffer

Reagent	Final concentration	Amount
PBS	N/A	2000 mL
Tween 20	0.05%	1 mL
**Total**	**N/A**	**2001 mL**

Store well closed at room temperature; store for up to 1 year.

## Step-by-step method details

### Pre-coating the culture flask or plates for HUVECs/HAECs

**Timing: 1 h**

1x Phosphate-buffered saline (PBS).

1% gelatin solution in sterile filtered water.

1. If using HAECs/HUVECs, the cell culture vessels need to be pre-coated with 1% gelatin (problem 1):
  a. Add 4 mL 1% gelatin solution to T75 cell culture flasks, 1 mL or 0.5 mL per well on 6 well plates or 12-well plates, respectively.
  b. Carefully spread the gelatin by moving the flask or plate in a cross-like pattern without creating bubbles until the entire bottom is coated with gelatin.
  c. Remove (≈3.5 mL) excess gelatin without leaving any bubbles.
  d. Put the culture flask and plate at 37°C for 30 min.
  e. Remove the excess gelatin once again (as much as possible without altering the coating (About few hundred microliters)) and leave the vessels under the hood for complete drying (for 30 min).
  f. Rinse the vessels with 1 mL or 0.5 mL of sterile PBS per well for 6 well plates or 12-well plates respectively before adding cell suspension.

***Note:*** If the cell culture flasks are closed airtight and sealed with parafilm, they can be stored at 4°C for a maximum of 3 days.

***Note:*** “incubator” refers to an incubator with 37°C, 5% CO_2_, and 95% relative air humidity.

***Note:*** When using a suction pump, carefully remove the gelatin excess without touching the bottom.

2. Calculate the needed culture surface area according to the plating density (see Table 1). Prewarm any needed media for the planned step in a 37°C water bath including:
  a. VSMCs:
    i. Smooth muscle cell growth medium (SMGM).
  b. HAECs/HUVECs:
    i. Endothelial cell growth medium (ECGM).
  c. 1x Phosphate-buffered saline (PBS).
  d. 1% gelatin solution.

**Table 1:** Medium requirements

	Prepared total amount of medium	Required amount of medium per well/flask	Suggested amount of VSMCs per plate/flask	Suggested amount of HAECs/HUVECs per plate/flask
75 cm^2^ culture flask	14 mL	14 mL	500 000 cells	500 000 cells
6-well plate	12.5 mL	2 mL	360 000 cells	480 000 cells
12-well plate	12.5 mL	1 mL	330 000 cells	450 000 cells
24-well plate	12.5 mL	0.5 mL	330 000 cells	450 000 cells
48-well plate	16 mL	0.3 mL	300 000 cells	450 000 cells
96-well plate	16 mL	0.15 mL	280 000 cells	400 000 cells
3. Label the cell culture flasks and plates with the cell type, the passage and date.

### Beginning a cell culture with cryopreserved vascular cells

**Timing: 45 min**

***Note:*** Cryopreserved cells should be stored at ‒196°C in a liquid nitrogen tank. Short- or long-term storage at ‒20°C will result in a severe decrease in cell viability, and long-term storage at ‒80°C is not sufficient for cell preservation.

4. Add 13 mL SMGM or ECGM to a 75 cm^2^ cell culture flask or a precoated 75 cm^2^ cell culture flask for culturing SMCs or HAECs/HUVECs respectively. Allow the culture vessel to warm up and equilibrate in an incubator for at least 30 min.
5. Take a cryovial containing the cells from the liquid nitrogen tank and immediately put it into a 37°C water bath for 2 min.

***Note:*** For safety reasons, when removing the cryovial from the liquid nitrogen tank, twist the cap partially open to relieve pressure and then retighten. Omitting this step may cause over pressurization of the cryovial during thawing, resulting in an explosion, body damage, and cell lost.

6. Wipe the cryovial and disinfect it with 70% Ethanol before opening it in a sterile bench.
7. Resuspend the cells by gently pipetting with a 1000 μL pipette.
8. Dispense the cells carefully in the respective prewarmed 75 cm^2^ cell culture flask, loosely tighten the cap.

***Note:*** Make sure that the cap for the flask is designed for sterile air exchanges.

9. Gently move the flask in cross-like pattern to distribute the cells homogenously.
10. Return the flask to the humidified 37°C incubator with 5% CO_2_.
11. Refresh the SMGM or ECGM the following day to remove the dimethyl sulfoxide (DMSO) residue in the freezing medium and any unattached cells.

***Alternatives:*** Transfer the cryovial content to a 15 mL tube and increase the volume with PBS or medium and centrifuge at 350 *g* for 5 min. Discard the freezing medium containing DMSO. Resuspend the cells in the respective medium prior to adding to the cell culture flask. This allows skipping step 8 but causes some cell loss due to the centrifugation. However, based on experience, the cells show a better performance when they are seeded directly.

12. Replace the medium after every two to three days until the confluency reaches up to 80%–90% for subcultivation.

***Note:*** DMSO in a high concentration can have impact on cell growth and viability.

### Subcultivation of vascular cells

**Timing: 1.5 h**

Before starting.

 Prepare the reagents and prewarm at 37°C.

 SMGM or ECGM.

 Detachment solution.

 Detachment stop medium.

 1x PBS.

 1% gelatin solution.

**Figure 1:**
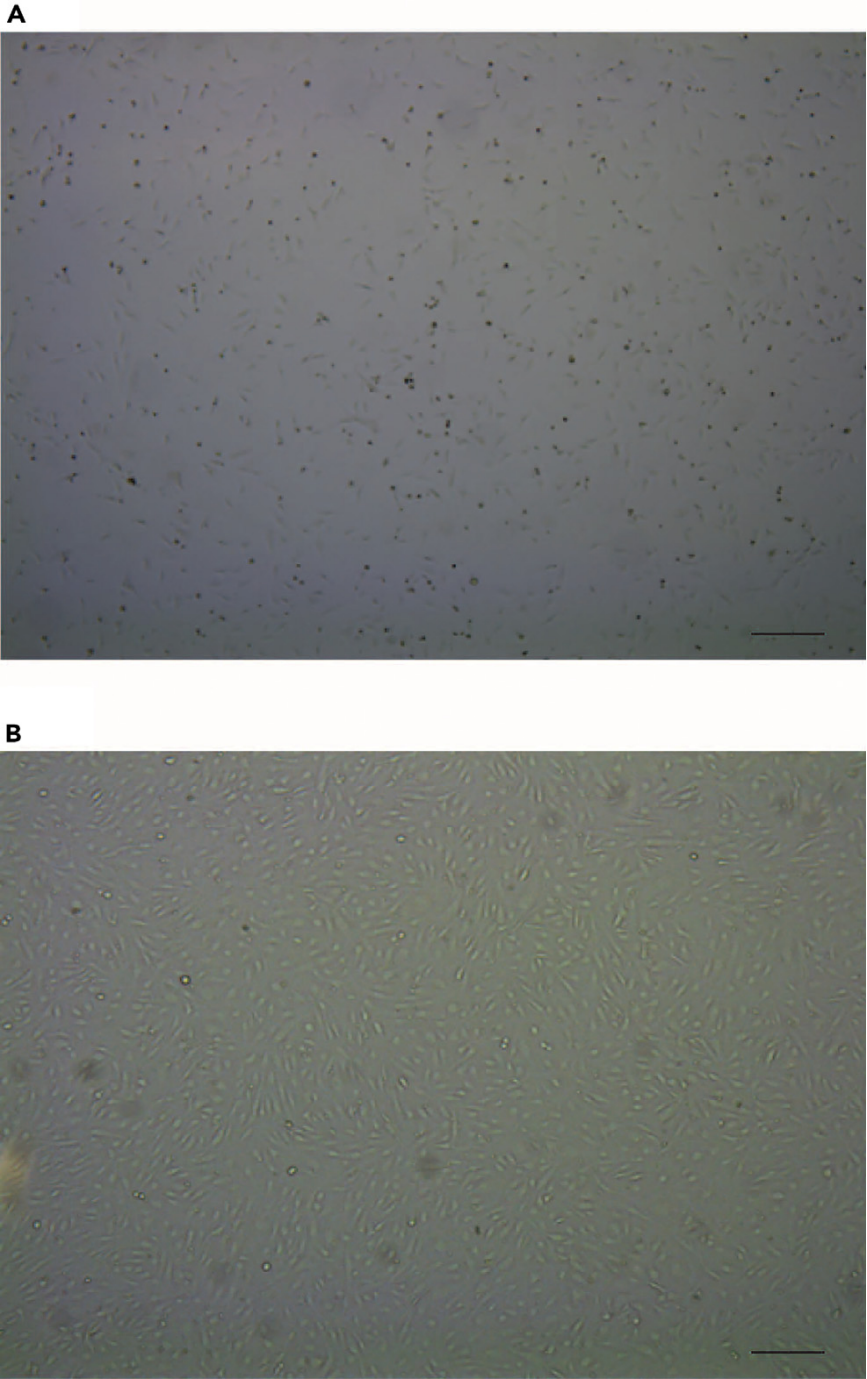
Morphological observation and identification of HUVECs Light microscopy of HUVEC density 24 h after seeding (A) and when reaching about 80% density (B). HUVEC were cultured in EBM-2 growth medium. In 1% gelatin coated vessels. The photos were taken under light microscopy with 4x magnification object.

**Figure 2:**
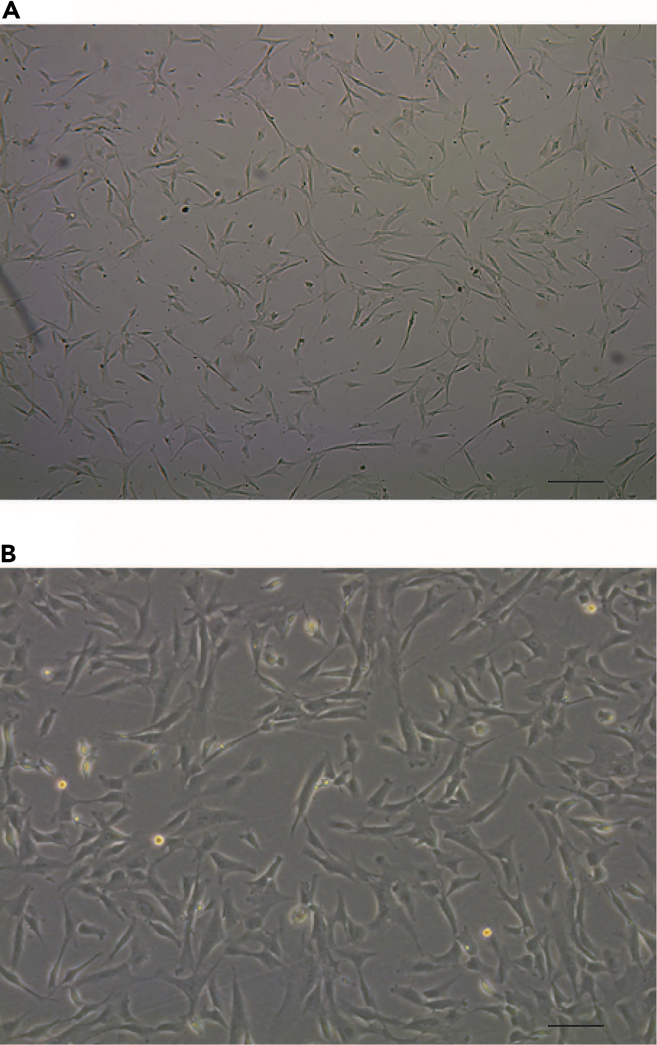
Morphological observation and identification of VSMCs Light microscopy of VSMC density 24 h after seeding (A) and when reaching about 70% density (B). VSMCs were cultured in M231 medium supplemented with SMCs growth supplement. The photos were taken under light microscopy with 4x (A) and 10x (B) magnification object.
14. Add 5 mL Detachment solution per 75 cm^2^ cell culture flask and place it into the incubator for 2–3 min.
15. Examine the cells under microscope and control cell detachment regularly. When the cells start detaching, slowly tap the side of the flask to loosen the remaining cells (problem 2).
16. Immediately add 5 mL Detachment stop medium.

***Note:*** FBS in the Detachment stop medium is essential for neutralizing the effect of Accutase or Trypsin.

***Note:*** Following thawing of the cryopreserved cells, proliferation might be delayed for 1–2 days (lag-phase). Afterwards, the cells will begin to proliferate logarithmically (log-phase). Due to their phenotypic plasticity, it is mandatory to avoid over confluency. The cells should be kept in the log-phase, and subcultivation should be considered when reaching about 70%–80% confluence (Figures 1A, 1B, 2A, and 2B).

13. Remove the cell culture medium and carefully rinse the cells twice with 5 mL of prewarmed PBS, swirling the liquid around inside the cell culture flask or plate before aspirating and discarding it.

17. Transfer the cell suspension to a new 50 mL falcon tube.
18. Wash out the remaining cells by adding 5 mL PBS and rinsing in all areas of the flask.
19. Resuspend the cells to make a homogenized suspension.
20. Count the cells in a Neubauer chamber under a microscope or using another available cell counter device.

***Note:*** A cell culture flask of 90% confluence consists of approximately 2.5 × 10^6^ cells (problem 3).

21. Centrifuge the tube at 350 *g* for 8 min at room temperature.
22. Aspirate and discard the supernatant and resuspend the cell pellet in 1 mL of appropriate growth medium (problem 2).
23. Calculate required number of the cells and make a cell suspension with desired cell concentration and amount of SMGM or ECGM, according to the recommended seeding density (Table 1).

***Note:*** Carefully resuspend the cells by gently pipetting up and down to make a single cell suspension.

***Note:*** The number of cells for seeding can be adjusted based on the proliferative abilities and thereby the time to 80% confluence of the cell type and according to donor variability.

24. Gently move the plate or flask in a small cross-like pattern to distribute the cells evenly (problem 2).

***Note:*** Check the cells under a microscope, and make sure that cells are distributed equally across the well. Uneven cell distribution may have a big impact on the experiment results.

25. Place the cell culture plates in an incubator and change the media every two to three days.

### Priming

**Timing: 2 days**

**Figure 3:**
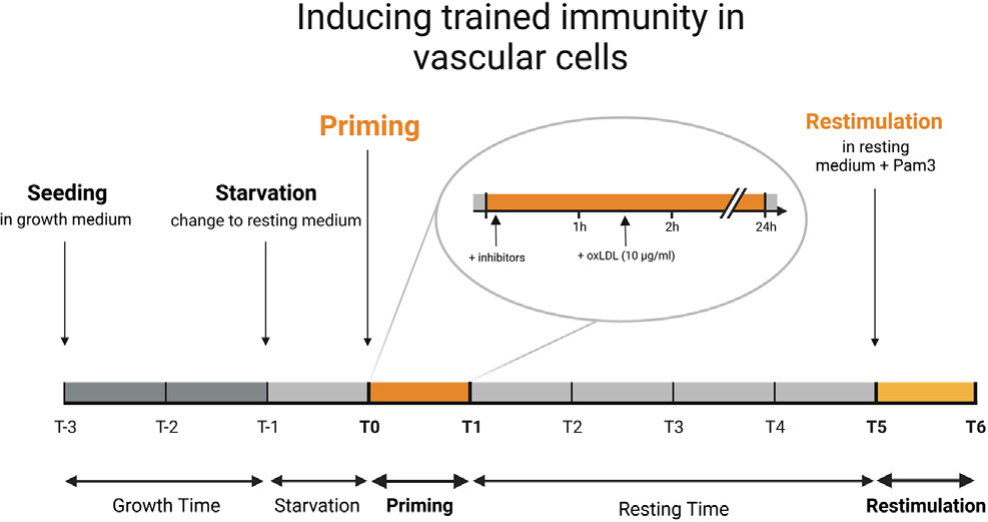
Representation of the presented protocol for induction of trained immunity in vascular cells Timeline in days.

In this step, the cells are primed with different stimuli like oxLDL (10 μg/mL) or BCG (5 μg/mL). Priming induces changes in the cell metabolism and the epigenetic profile, thereby enhancing the proinflammatory response of the cells upon secondary restimulation with lipopeptide tripalmitoyl-S-glycerylcysteine (Pam3Cys4) (5 μg/mL)^1^^,^^2^^,^^6^ (Figure 3).

26. Check the cells under the microscope, assuring a lack of contamination. If the confluency is approximately 70%, proceed to the next step.
27. Change the cell culture medium to resting medium for a 24 h starvation period prior to priming (1 mL per well on a 12-well plate).
  a. Discard the old medium using a suction pump or pipette.
    **CRITICAL:** Avoid touching the cells with pipette tips or letting them dry out. If using a suction pump, avoid disrupting the cell layer by sucking the well completely dry.
  b. Add fresh medium by pressing the tip of the pipette against the wall and letting it run in slowly to avoid mechanical cell damage.
    **CRITICAL:** High pipetting pressure can damage the cell layer. Gently add the medium and avoid disrupting the cells.
28. When using pharmacological inhibitors in an experiment, add them at least 1 h prior to adding the priming reagent (problem 4).

**CRITICAL:** When using an organic solvent such as DMSO, prepare corresponding controls.

29. Add the required dilution of the priming reagent (e.g., 10 μg/mL oxLDL), if necessary dilute in SMRM or ECRM. Shake the cell culture plate carefully to ensure equal distribution of oxLDL and return it to the incubator.
30. After 24 h change the cell culture medium to fresh resting medium, thereby ending the priming and starting the resting time.

### Resting period

**Timing: 4 days**

The vascular cells are incubated in resting medium for four days, before harvesting them for subsequent analysis or restimulating them with a secondary unspecific inflammatory stimulus.

31. Refresh the SMRM or ECRM after every two or three days (problem 5).

### Harvesting for further analysis

**Timing: 6 h to 24 h**

The establishment of the innate immune memory is evaluated in this step. The cells are either lysed for metabolic or epigenetic studies or restimulated with a secondary unspecific inflammatory stimulus, provoking inflammatory responses in primed cells in comparison to the control group. Enhanced inflammatory responses are checked by expression of inflammatory genes, such as IL-6 and IL-8 by real-time quantitative PCR (qPCR) or cytokine/chemokine production by enzyme-linked immunosorbent assay (ELISA).

Restimulation with a TLR agonist.

32. Prepare a restimulation medium by adding 5 μg/mL of Pam3Cys4 (a TLR2 agonist) or 10 ng/ml of LPS (TLR4 agonist) to the appropriate resting medium for the cell type.
33. Change the cell culture medium to the Pam3Cys4 containing medium.

**CRITICAL:** Avoid touching the cells with pipettes or letting them dry out. If using a suction pump, avoid disrupting the cell layer by sucking the well completely dry.

34. 4–6 h after restimulation the cells can be lysed with an appropriate lysing buffer for RNA isolation and/or qPCR for gene expression analysis. The lysate can be processed immediately or stored at ‒80°C until it is used.
35. 24 h after restimulation collect the supernatants and perform ELISA immediately or freeze the samples in ‒20°C for later.

***Note:*** Only one of the steps explained under 3. and 4. can be performed with one single well.

***Note:*** Different treatments may have varying impacts on the cell density. Therefore, ELISA data should be normalized to the number of alive cells in the well. For adherent cells, staining with 1 μM Calcein AM is recommended.

36. Add the corresponding volume of 1 μM Calcein AM in warm 1% BSA in PBS (pH 7.4).
37. Incubate the plates at 37°C for 20 min.
38. Measure the fluorescence intensity by using filters for FITC/Alexa Fluor 488 (problem 6).

### ELISA

**Timing: 2–3 days**

The soluble inflammatory cytokines are analyzed in the supernatant samples using enzyme-linked immunosorbent assays (ELISA) according to the manufacturers' instructions. The goal of this setup is to show enhanced cytokine production in the cells trained with inflammatory stimuli.

Antibody coating buffer: 1x PBS (pH 7.4).

**CRITICAL:** Coating buffer must not contain any carrier protein such as BSA or PBS. Antibodies may unspecifically react to the carrier proteins and dramatically reduce coating efficiency.

Reagent Diluent: 1x PBS supplemented with 1% BSA (pH 7.4).

***Alternatives:*** BSA could be replaced with FBS.

Washing buffer: 1x PBS supplemented with 0.05% Tween-20 (pH 7.4).

Stop solution: 1 M Hydrochloric acid (HCl) or 1 M Sulfuric acid (H_2_SO_4_).

39. Day 1: Coating the plates:
  a. Prepare the coating buffer with appropriate volume of capture (primary) antibody according to the manufacture recommendation (R&D). Working concentration of capture antibody for IL-6 and IL-8 are 2 and 4 μg/mL respectively.
    **CRITICAL:** Briefly centrifuge the capture antibody before use.
  b. Coat the ELISA plate with suggested volume/well of the coating buffer.
  c. Seal and incubate the plate overnight at room temperature.
    ***Note:*** The ELISA plates have high binding properties; the hydrophilic surfaces are optimized to bind a high amount of antibodies.
40. Day 2: Blocking:
  a. Discard the coating solution and wash the plate 3x with 150 μL/well washing buffer.
  b. Tap the plate on a paper towel to remove any residual buffer.
  c. Block the wells with 150 μL/well 1x Reagent Diluent and incubate for at least 1 h at room temperature.
41. Preparing the standards and adding the samples:
  a. Prepare the standard in duplicates in a serial dilution (11 standards + blank).
  b. Wash 3x with 150 μL/well washing buffer. The concentration of the highest standard for IL-6 and IL-8 are 1200 and 4000 pg/mL respectively.
    ***Note:*** Do not let the wells dry up completely.
  c. Add the required dilution of the standards in the wells.
  d. Add proper volume of the samples with appropriate dilution (diluted in assay diluent) to the well. Seal the plate and incubate at room temperature for 2 h on the shaker.
    **CRITICAL:** It is very important to find an appropriate dilution of the samples per well. This can vary and depend on the cell type and cell culture condition. For instance, for detecting IL-8 concentration after Pam3Cys4 restimulation in SMCs, it is necessary to dilute the sample between 20‒50 times.
    ***Note:*** Incubation of the plates containing the samples can be optimized. For detecting IL-1β, VEGF1, or other targets with low concentration, we recommend to incubate the plates containing supernatant overnight at 4°C.
42. Adding the detection antibody:
  a. Prepare the required volume of detection (secondary) antibody. The concentration of the detection antibodies for IL-6 and IL-8 are 50 and 20 ng/mL respectively.
    **CRITICAL:** Briefly centrifuge the antibody before use.
  b. Flip the plates and wash 3x with 150 μL/well washing buffer.
  c. Add an appropriate volume of the detection antibody/well. Seal the plate and incubate 2 h at room temperature on a shaker.
43. Adding Avidin-HRP (-horseradish peroxidase):
  a. Prepare required volume of Avidin-HRP according to the manufacturers guidelines.
  b. Flip the plates and wash 3x with 150 μL/well washing buffer.
  c. Blot the plate on a paper towel to remove any residual buffer.
  d. Add 100 μL/well Avidin-HRP to all wells. Put the plate in the dark with a lid on and incubate at room temperature for 20–30 min.

**CRITICAL:** Avidin-HRP must be kept in the dark, avoid incubating the solution or the plates in the light.

44. Adding HRP substrate (3,3,5,5- tetramethylbenzidine (TMB):
  a. Prepare required volume of HRP substrate.
  b. Wash at least 4x with 150 μL/well washing buffer.
  c. Add 100 μL substrate/well. Incubate the plates at room temperature for 10–20 min in the dark.

***Note:*** Check the plates repeatedly in order to prevent saturation of color development.

45. To terminate the HRP/TMB reaction, add 50 μL of the stop solution into the wells.

**Caution:** Wear protective gloves, clothing, and eye and face protection.

46. Measure the color absorbance at 450 nm with a plate reader.

## Expected outcomes

**Figure 4:**
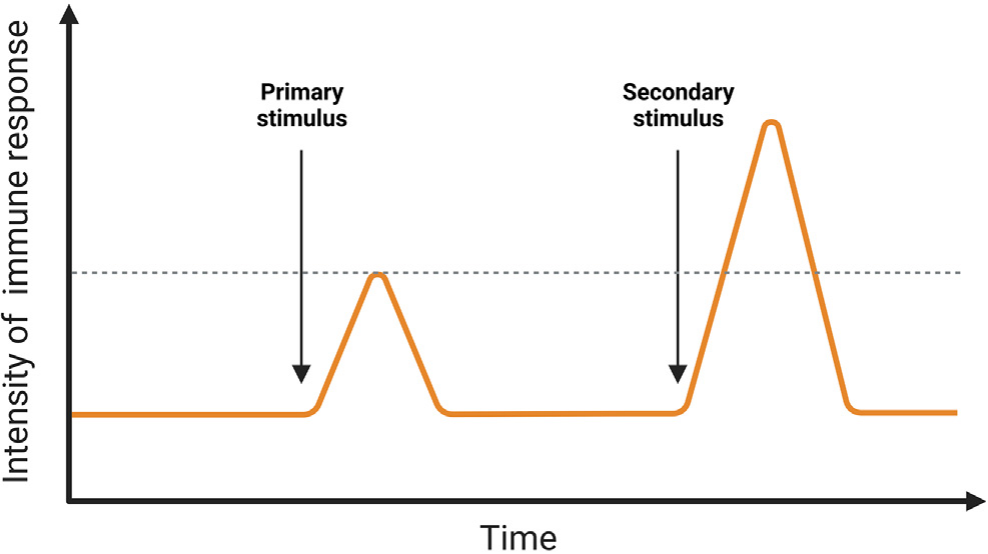
Expected results of inducing innate immune memory The primed cells are expected to show enhanced immune reaction in response to a secondary stimulus.

**Figure 5:**
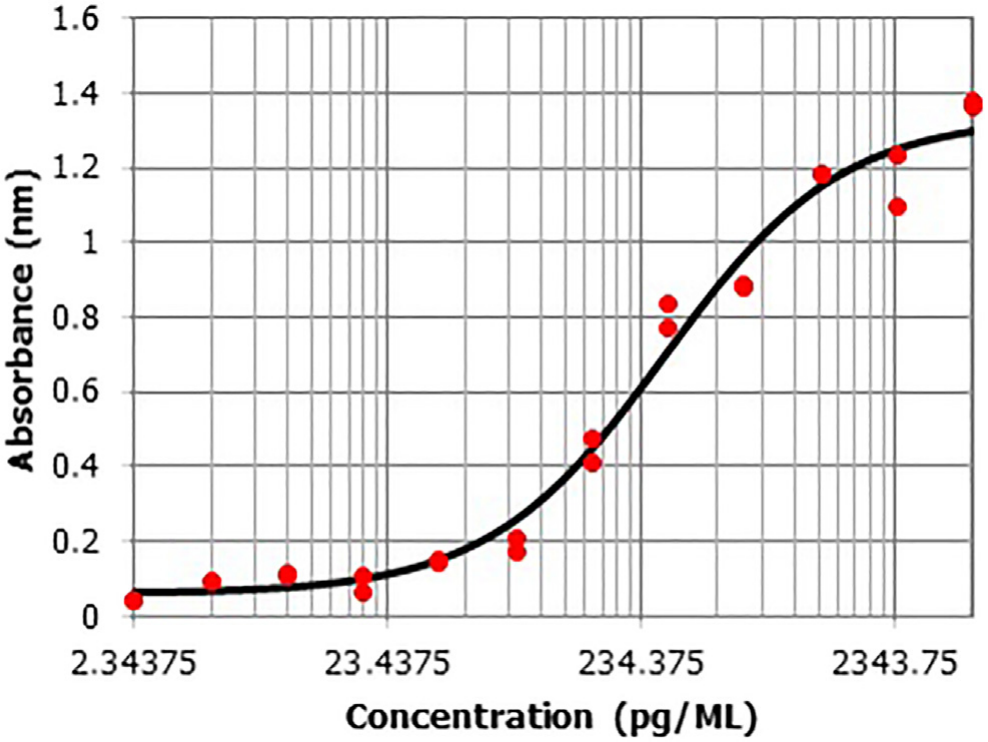
Example of a standard curve for analyzing ELISA data

**Figure 6:**
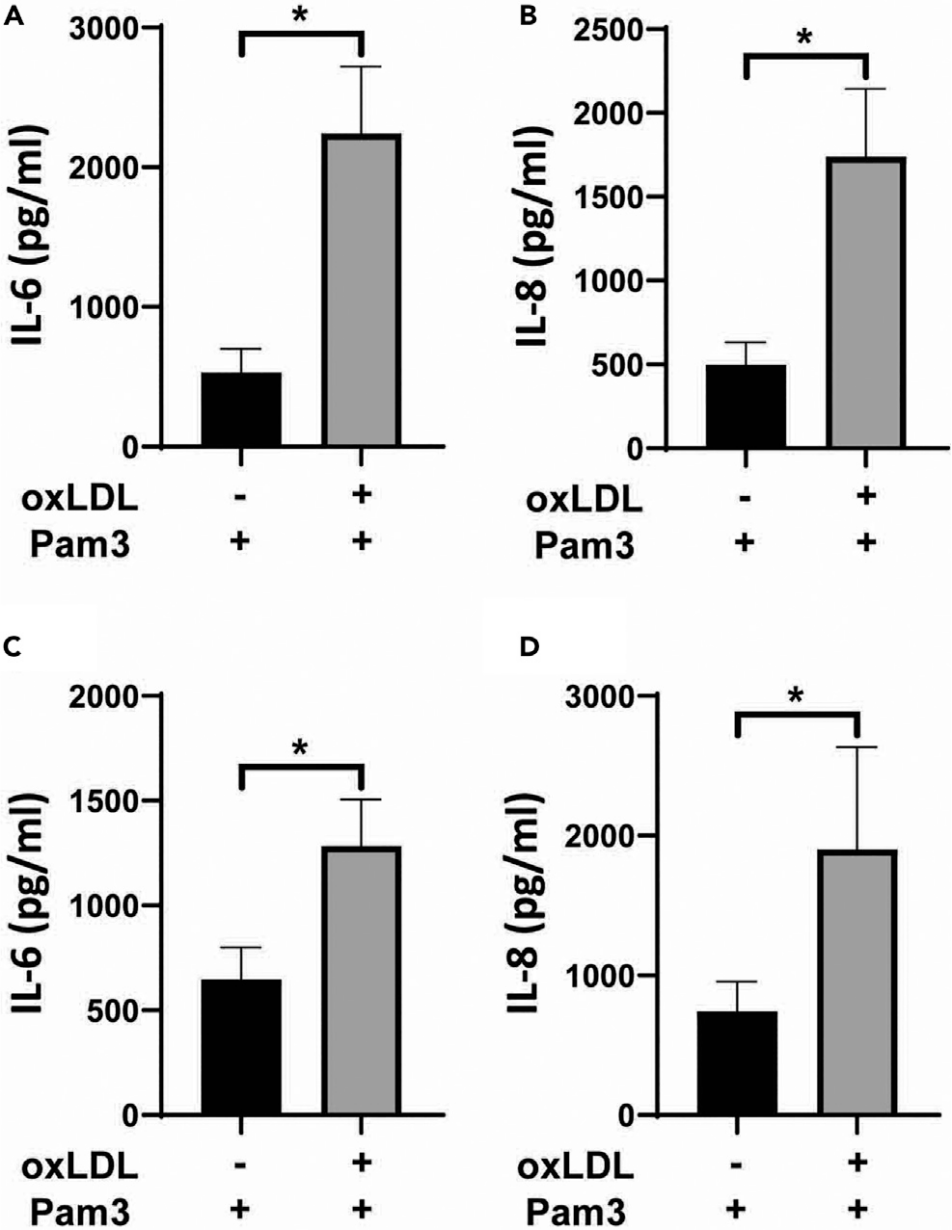
oxLDL priming induces a proinflammatory phenotype in HUVECs and VSMCs oxLDL induced trained immunity in VSMCs (A, B) and HUVECs (C, D). VSMCs and HUVECs were primed with 20 μg/mL of oxLDL for 24 h. The medium was changed and the cells were rested for 4 days. Then, the cells were restimulated with 5 μg/mL of Pam3Cys4 for 24 h and IL-6 and IL-8 concentration were measured using ELISA. Graphs represent mean values ± SD. ∗p < 0.05.

The goal of this protocol is, to induce and analyze an innate immune memory in vascular cells. Therefore, the expected result is an enhanced immune response in primed VSMCs and HAECs/HUVECs upon secondary stimulation such as Pam3Cys4 (Figure 4, Figure 5, Figure 6A–6D). This is represented in terms of increased concentrations of inflammatory cytokines such as IL-6 and IL-8 in comparison to a control group lacking the primary stimulus. These can be measured via ELISA or real-time qPCR.

## Quantification and statistical analysis

**Figure 7:**
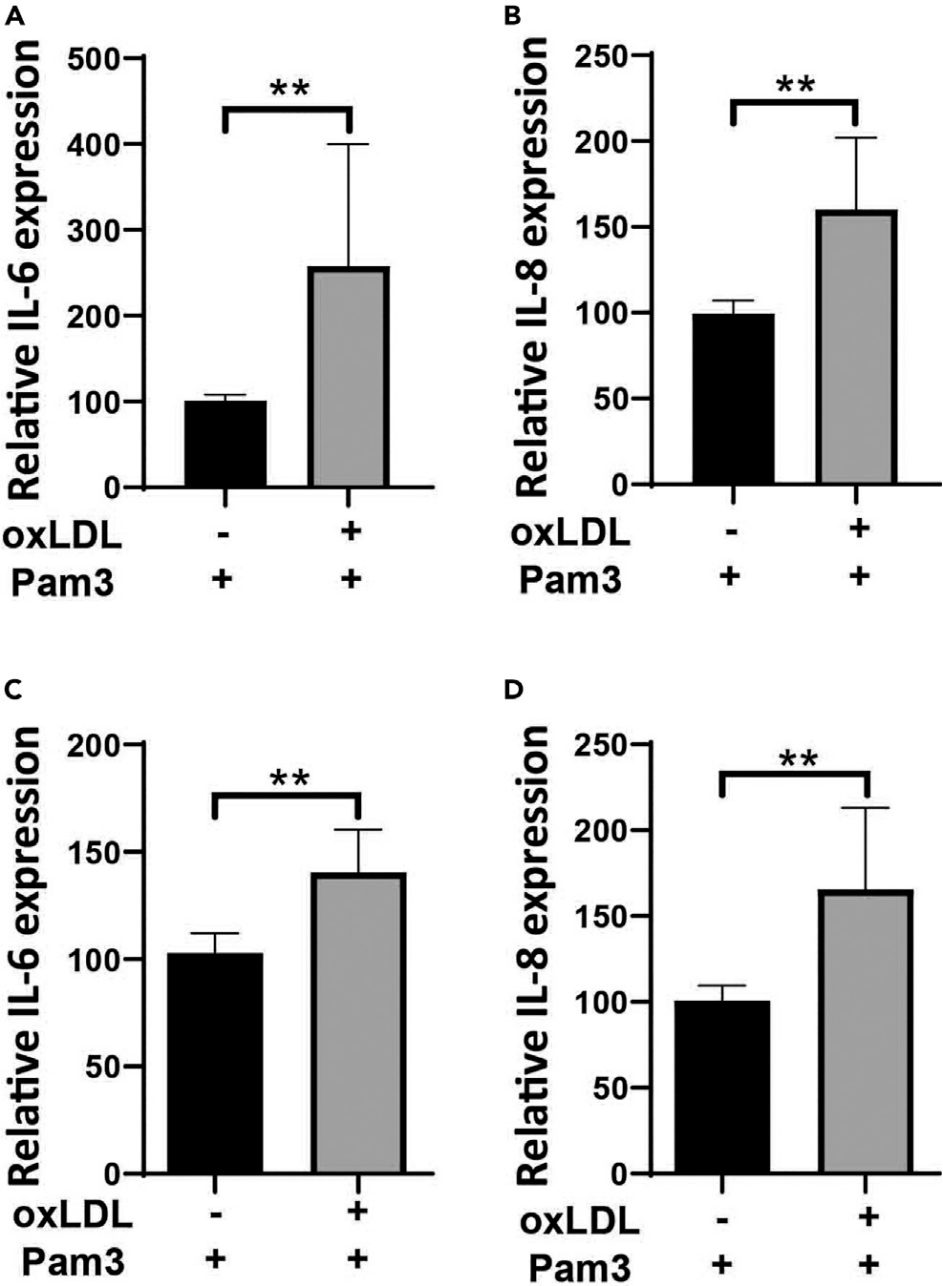
oxLDL priming induces upregulation of inflammatory genes in HUVECs and VSMCs oxLDL induced innate immune memory in VSMCs (A, B) and HUVECs (C, D). VSMCs and HUVECs were primed with 20 μg/mL of oxLDL for 24 h. The medium was changed and the cells were rested for 4 days. Then, the cells were restimulated with 5 μg/mL of Pam3Cys4 for 6 and lysed for RNA isolation. IL-6 and IL-8 mRNA expression were measured using qPCR.Graphs represent mean values ± SD. ∗p < 0.05.

For processing raw ELISA data, any software, which creates four parameters logistic regressions such as WorkOut 2.5 by Dazdaq or the MyAssays online program can be used (Figure 7).

***Note:*** The response and level of cytokines may differ among the donors. Basic level of IL-6 and IL-8 usually is higher in vascular cells in comparison to monocytes.

## Limitations

This is an *in vitro* approach for the induction of trained immunity and may not reflect the complexity of *in vivo* experiments.

The protocol is optimized for human vascular cells using limited donors. It is applicable to other cell types and donors. However, it may need further adjustments and optimization regarding timing and concentrations of chemicals.

## Troubleshooting

### Problem 1

No coating or partial coating of 1% gelatin.

### Potential solution

When coating the plates with 1% gelatin, creation of bubbles or solid particles may cause uneven coating and results in not homogenous confluence of the cells. Slowly shake the flasks or the plates. Make sure that gelatin solution covers the surface area completely. Make sure that no solid particles or bubbles are left after the coating. Monitor the coating quality by slightly shaking the flask or plate. Pipet out any solid particles and the excess gelatin solution over the bubble until it has disappeared.

### Problem 2

Suboptimal cell distribution after sub-cultivation of VSMCs or endothelial cells.

### Potential solution

When adding the Detachment solution, any serum left in the well/flask might inactivate the solution and lead to uneven detachment results.

In addition, leaving cells in Detachment solution for too long when subcultivating, may damage them and lead to cell clumping. Fast detachment of the cells may lead to cell damage. Moreover, the same might occur to already detached cells if the overall process takes > 5 min. Reduce or increase the amount of the Detachment solution used, to detach the cells accordingly. Remember to adjust the amount of detachment stop medium as well and control multiple times for cell detachment, in order to stop the reaction, before causing severe damage to the cells.

After centrifugation, resuspend the cells in less medium. Clumping can be prevented by pipetting a little more vigorously and repeatedly, yet this may result in cell damage. Furthermore, when distributing cells mechanically in the wells after seeding, less movement is sometimes better. Moving plates in circular patterns may lead to uneven distribution of the cells. Move your well plates slowly and carefully after seeding, e.g., when putting them into the incubator and assure that the shelves in the incubator are level, if experiencing a drift of cells towards one side of the wells.

### Problem 3

Problem with cell detachment, significant number of cells left in the flask.

### Potential solution

Make sure that the Detachment solution is prewarmed to 37°C, as the detachment process is not efficient when using cooled solutions.

Any serum left in the flask might inactivate the Accutase contained in the Detachment solution. Always wash at least two times with PBS before adding the Detachment solution. If there are cells left in the flask after the detachment, the cycle of adding Detachment solution and stopping the reaction can be repeated. Rather repeat the cycle than incubating the cells longer with Accutase, as this might damage the already detached cells. If doing so, remember to wash the flask again with PBS before adding the Detachment solution, to minimize serum left from the Detachment stop medium.

### Problem 4

Data in between multiple runs is deviating.

### Potential solution

This might be due to basic donor variation if using multiple cell lines. Cells may also behave differently, when used in higher passages. For comparability, we recommend to use the cells up to passage 7.

Another reason may be pipetting mistakes.

Firstly, check if the pipette tip is attached tightly. Differently attached tips might lead to different volumes pipetted every time the tip is changed, e.g., when air is aspirated because the tip is not firmly attached. Be extremely aware of this issue, when using a multichannel pipette.

Secondly, avoid pipetting small volumes. When pipetting volumes < 2 μL, small variations and droplets stuck to the tip can affect heavily on the results. Therefore, dilute smaller volumes in cell culture medium before adding to the wells.

Lastly, most pipette tips feature scale ring marks according to different volumes, which facilitate the control of the aspirated volume.

Bacterial or fungal contamination may lead to imprecise results. Therefore, regularly scan your well plates with an optical microscope, using magnifications such as 10x or 20x to spot any contamination.

### Problem 5

Cell detachment and cell death after adding chemicals to the cell culture.

### Potential solution

Firstly, assure that the concentrations of the solutions are correctly calculated. If encountering cell detachment in serious extents, it is important to titrate the chemicals to determine the right concentration. We recommend to performed cell toxicity assays such as LDH assays.

In addition, mechanical handling of the cells such as forcefully pipetting when adding medium, may cause cell detachment.

Furthermore, starting the experiment with too low confluency may lead to cell detachment under mechanical stress.

### Problem 6

Results show little to no difference between the groups receiving priming and the control.

### Potential solution

**Figure 8:**
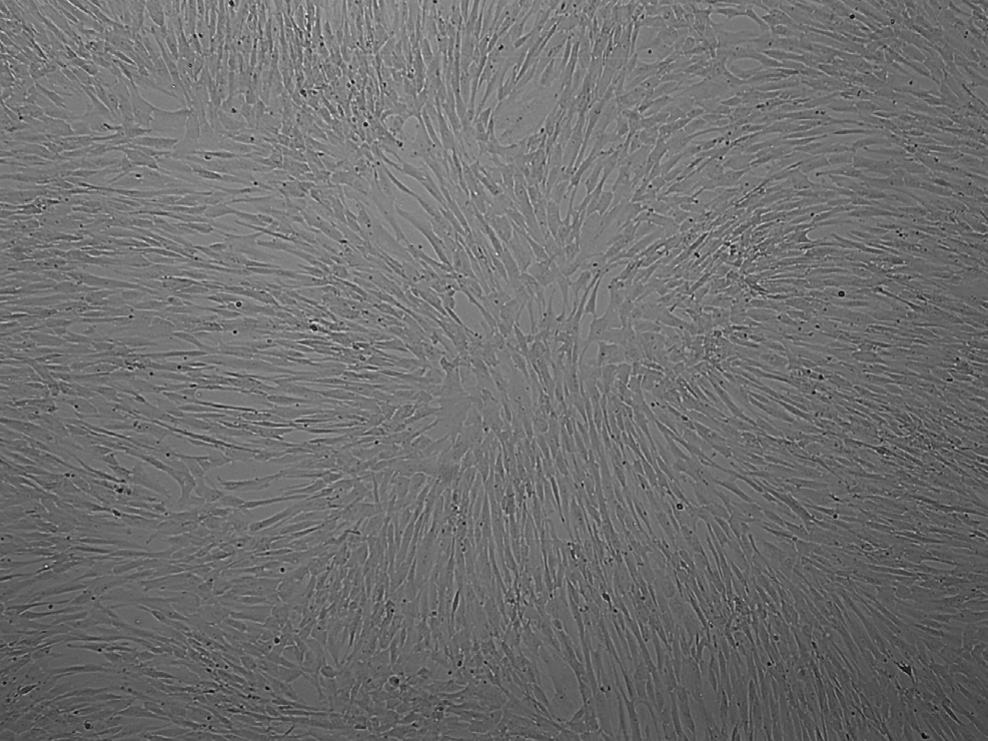
Over confluent VSMCs show low priming effect

There may be multiple causes for this, as VSMCs react quite strongly to external and internal stimuli. Over confluent cells show very low or no priming effect (Figure 8). Make sure that the cells are 70%–80% confluent at the time priming. In addition, unequal distribution of cells can also influence priming efficiency (see problem 2). Concentration of priming compound and secondary stimuli should be optimized. Using an old batch of priming solution may cause no effect. Prepare a fresh priming material.

## Resource availability

### Lead contact

Further information and requests for resources and reagents should be directed to and will be fulfilled by the lead contact, Yahya Sohrabi (yahya.sohrabi@ukmuenster.de).

### Technical contact

The technical contacts for this protocol are Dennis Schwarz (dennis.schwarz@ukmuenster.de) and Sina MM. Lagasche (sina.lagasche@gmail.com).

### Materials availability

This study did not generate new and unique reagents.

### Data and code availability

The published article includes all datasets generated or analyzed during this study.
